# Glycosylation Site Alteration in the Evolution of Influenza A (H1N1) Viruses

**DOI:** 10.1371/journal.pone.0022844

**Published:** 2011-07-28

**Authors:** Shisheng Sun, Qinzhe Wang, Fei Zhao, Wentian Chen, Zheng Li

**Affiliations:** Laboratory of Functional Glycomics, College of Life Sciences, National Engineering Research Center for Miniaturized Detection System, Northwest University, Xi'an, People's Republic of China; Center for Disease Control and Prevention, United States of America

## Abstract

Influenza virus typically alters protein glycosylation in order to escape immune pressure from hosts and hence to facilitate survival in different host environments. In this study, the patterns and conservation of glycosylation sites on HA and NA of influenza A/H1N1 viruses isolated from various hosts at different time periods were systematically analyzed, by employing a new strategy combining genome-based glycosylation site prediction and 3D modeling of glycoprotein structures, for elucidation of the modes and laws of glycosylation site alteration in the evolution of influenza A/H1N1 viruses. The results showed that influenza H1N1 viruses underwent different alterations of protein glycosylation in different hosts. Two alternative modes of glycosylation site alteration were involved in the evolution of human influenza virus: One was an increase in glycosylation site numbers, which mainly occurred with high frequency in the early stages of evolution. The other was a change in the positional conversion of the glycosylation sites, which was the dominating mode with relatively low frequency in the later evolutionary stages. The mechanisms and possibly biological functions of glycosylation site alteration for the evolution of influenza A/H1N1 viruses were also discussed. Importantly, the significant role of positional alteration of glycosylation sites in the host adaptation of influenza virus was elucidated. Although the results still need to be supported by experimental data, the information here may provide some constructive suggestions for research into the glycosylation of influenza viruses as well as even the design of surveillance and the production of viral vaccines.

## Introduction

Influenza virus can cause occasional pandemics and seasonal epidemics in humans [1]. The great threat and high frequency of variation of influenza viruses make the study of their evolutionary mechanisms for host adaptation an urgent necessity in order to predict and prevent the outbreak of potential new influenza pandemics in the near future [2]. The H1N1 virus was an ideal model for this purpose due to its long history of association with humans, as well as co-circulation with the H3N2 virus in swine and avian hosts [3], [4], [5].

The genome of the influenza A virus encodes 11 proteins. However, only hemagglutinin (HA) and neuramidinase (NA) undergo *N-*linked glycosylation, and no observation of *O-*linked glycosylation has been reported [6], [7]. HA and NA are critical determinants of the host specificity, virulence and infectivity of the influenza A virus. Glycosylation of HA and NA can affect the host specificity, virulence and infectivity of an influenza strain either directly, by changing the biological properties of HA and NA [8], or indirectly, by attenuating receptor binding [9], [10], [11], [12], [13], masking antigenic regions of the protein [8], [14], [15], [16], [17], impeding the activation of the protein precursor HA0 via its cleavage into the disulfide-linked subunits HA1 and HA2 [18], [19], [20], regulating catalytic activity or preventing proteolytic cleavage of the stalk of NA [2], [21], [22]. *N-*linked glycosylation sites generally fall into the *N-*X-S/T sequence motif (sequon) in which X denotes any amino acid except proline [23]. The number and distribution of the *N-*glycosylation sites over the viral proteome can therefore be computationally determined by scanning the sequences for these sequons [24], [25].

Previous reports have shown that the seasonal H1N1 viruses possess more *N-*glycosylation sequons in their HA sequences than the 1918 H1N1 strain (A/South Carolina/1/18) [17], [25], [26], [27]. Two highly conserved glycosylation sites (Asn 142 and Asn 177) in the receptor binding domain A (RBD-A) of HA in the seasonal strains (represented by A/New Caledonia/20/1999) endow the seasonal virus with resistance to antibodies directed against both of the pandemic strains from 1918 and 2009 [26], [28]. Because these two glycosylation sites are absent in both pandemic strains, cross-neutralization can occur between the two temporally distant pandemic influenza viruses, although both are insensitive to antisera to 1999 NC. In addition, the focus of the immune response to HA in both seasonal and pandemic strains could be selectively changed by the removal or addition of glycans on glycosylation sites 142 and 177 [26], [28]. Das and coworkers [17] also reported the influence of *N-*glycosylation on HA evolution by employing bioinformatics tools. In addition, Wu and coworkers [22] showed that the distinct *N-*glycan profiles of NA from the 1918 pandemic influenza virus might cause viral resistance to proteinase digestion as well as high infectivity. Protein glycosylation is beginning to be recognized as one of the important ways influenza viruses evolve [26], [29]. However, the alteration modes and laws of protein glycosylation in the evolution of influenza A/H1N1 viruses are not fully understood.

In this study, the patterns and conservation of the potential *N-*glycosylation sites in 2,773 full-length amino acid sequences of HA and 3,249 full-length amino acid sequences of NA for influenza H1N1 viruses were systematically analyzed by employing a series of bioinformatics tools. The alteration modes and laws of protein glycosylation sites in terms of the evolution of human influenza H1N1 viruses were discussed. And the significant role of positional alteration of glycosylation sites in the host adaptation of influenza virus was elucidated.

## Materials and Methods

### Protein sequence data of HA and NA from influenza A/H1N1 viruses

A total of 2,773 full-length amino acid sequences of HA and 3,249 full-length amino acid sequences of NA from influenza A (H1N1) viruses isolated from various hosts were downloaded from the influenza virus resource at the National Center for Biotechnology Information (NCBI) (http://www.ncbi.nlm.nih.gov/genomes/FLU/FLU.html) [30], [31] as of March 30, 2010. For evolutionary analysis, the human seasonal influenza H1N1 viruses were further divided into several groups according to their glycosylation site patterns. Before predicting the potential *N-*glycosylation sites of HA and NA, multiple-alignments of the HA and NA sequences were performed using the influenza virus sequence alignment tool available at the NCBI website (http://www.ncbi.nlm.nih.gov/genomes/FLU/Database/nph-select.cgi?go=alignment) [30]. Note that when the number of sequences used exceeds the maximum of 1000 that is allowed to run on the NCBI sequence alignment server, the sequences were separated into two or three groups. After alignments, they were merged into one file again for further analysis. Swine strains isolated from humans were not included in the human seasonal influenza viruses.

### Prediction and statistical analysis of potential *N-*glycosylation sites

Sequon Finder was used to predict *N-*glycosylation sites on HA and NA and to perform statistical analysis of the glycosylation site conservation among the viruses. Sequon Finder is a custom-made program that just simply finds all sequons (*N-*X-S/T, where X is not P) within protein sequences and supposes all of sequons as potential glycosylation sites. Then it will compute the percentages of the sequon appeared at each location in all protein sequences as the conservation of the potential glycosylation site among homologous proteins. The number of glycosylation sites was obtained from a single monomer of HA and NA and the locations of the glycosylation sites on HA and NA were numbered according to the full- length HA sequence of South Carolina/1/1918 and the full- length NA sequence of Brevig Mission/1/1918, respectively. The results of the statistical analysis were manually validated. The program is available upon request.

### Homology modeling, *in silico* protein glycosylation and visualization

To visualize and determine the positions of the glycosylation sites, the 3D structures of representative HA and NA proteins with different patterns of potential *N-*glycosylation sites in human influenza A (H1N1) viruses were generated using SWISS-MODEL (http://swissmodel.expasy.org/) [32]. The crystal structure of A/puerto rico/8/1934 HA (PDB code: 1RU7, http://www.rcsb.org) and A/California/04/2009 HA (PDB code: 3LZG) were used as the HA models of the human influenza H1N1 viruses before and after 2000, respectively. An influenza A (H5N1) NA (PDB code: 2hty) was used as the NA model. After homology modeling, glycans were added onto the potential *N-*glycosylation sites of HA and NA using the Glyprot Server (http://www.glycosciences.de/modeling/glyprot/) [33]. Complex glycan structures were selected for all accessible sites, and the terminal sialic acid residues were manually removed. All of the figures were generated and rendered using MacPyMOL [34].

## Results

### Comparison of glycosylation site patterns on HA and NA between human pandemic and seasonal influenza viruses

First, we compared the patterns of potential *N-*glycosylation sites for the HA and NA sequences of 1918 and 2009 pandemic human influenza A (H1N1) viruses as well as human seasonal influenza H1N1 viruses. Pandemic viruses are newly emerging viruses, and thus may be the origin of seasonal viruses. While human seasonal viruses originating from pandemic viruses may have undergone many changes in its antigenic structure (called antigenic drift) [1], [5]. Therefore, the comparison of seasonal to the 1918 and 2009 pandemics can speculate the overall trends of glycosylation site alteration in seasonal viruses.

The results showed, as had been reported previously [17], [25], [26], [27], that the seasonal viruses had more glycosylation sites on the head of HA and NA than the 1918 and 2009 pandemic viruses, and that two glycosylation sites (glycosylation sites 50 and 68) on the stalk of the NA in pandemic viruses likely be replaced by another two glycosylation sites (glycosylation site 44 and 70) in seasonal strains (Table 1, Table 2). Besides, there was also one more glycosylation site on both HA (glycosylation site 293) and NA (glycosylation site 386) of the pandemic 2009 strains than that of the pandemic 1918 strains. Note that one HA sequence and one NA sequence should still be able to represent glycosylation site patterns of HA and NA in pandemic 1918 virus, as pandemic 1918 viruses had originated from avian influenza viruses [5] and almost all avian influenza viruses possessed the same glycosylation site patterns on HA and NA as that of the pandemic 1918 virus (Table 1).

**Table 1:** The patterns and conservation of potential glycosylation sites on HA in different type of A/H1N1 influenza viruses.

Location	HA1																				HA2				
	Stalk		Side of head							Top of head					Side of head						Stalk				
	27	28	40	71	73	90	91	104	136	142	144	172	177	179	201	212	239	286	291	293	304	425	472	489	498
Human pandemic 1918 (1)	100	100	100					100													100				100
Human pandemic 2009 (808)	100	100	99.88					99.63	0.12					0.5						**97.65**	99.51		0.25	0.37	100
Human seasonal (1503)	100	100	99.67	91.62	0.2	0.6		98.34		89.49	6.59	6.65	94.48	0.6				13.97			99.6				99.87
1933 (6)	100	100	33.33		**50**					**66.67**				**33.33**				**100**			33.33				83.33
1934–1939 (11)	100	100	100								**27.27**							**100**			100				100
1940–1949 (13)	100	100	100					69.23			**61.54**	**15.38**		**53.85**				**100**			100				100
1950–1957 (10)	100	100	100			**80**		70		10	**80**	**80**	**60**					**80**			100				100
1977–1985 (83)	100	100	100			1.2		100			**96.39**	**98.8**	**98.8**					**100**			100				100
1986–1987 (9)	100	100	100					100		**100**		**88.89**	**88.89**					**100**			100				100
1988–1997 (72)	100	100	100	**91.03**				86.69		**95.83**			**94.44**					**76.39**			100				100
1998–2009 (1299)	100	100	99.92	**99.24**				100		**97.15**			**96.69**					1.92			99.85				100
Avian (100)	98	98	100	1				99					1							5	100				100
Swine (331)	99.7	99.7	100	2.11	0.3		0.3	85	0.91	1.8	0.3		1.21	10.88	2.42	13.6	0.6	0.91	**20.24**	**39**	79	0.3			100

The human seasonal H1N1 viruses were separated into eight groups according to their glycosylation site patterns. The conservation of potential glycosylation sites was shown as percentage (‘%’ had been omitted) and the glycosylation site alteration was highlighted in bold. The numbers of corresponding strains used for the analysis were given in the brackets.

**Table 2:** The patterns and conservation of potential glycosylation sites on NA in different type of A/H1N1 influenza viruses.

Location	Stalk						Head									
	44	50	58	63	68	70	88	146	235	341	365	386	398	434	449	455
Human pandemic 1918 (1)		100	100	100	100		100	100	100							
Human pandemic 2009 (702)		100	100	100	100		99.86	100	100			**99.29**				
Human seasonal (2032)	99.75	0.74	99.36	99.11	2.66	96.26	99.75	99.16	99.75		5.71	2.8		93.41		97.54
1933 (9)	**100**						100	22.22	100							
1934 (7)	**100**		100				100	28.57	100							
1935–1947 (19)	**89.47**	**73.68**	89.47	94.74	**89.47**		100	100	100		**63.16**					
1948–1979 (36)	**100**		100	100	**100**		100	100	100		**97.22**					**100**
1980–1985 (60)	**100**		100	100	1.67	**98.33**	100	100	100		**98.33**					**100**
1986–1987 (10)	**100**		100	100		**100**	100	100	100		**100**			**100**		**100**
1988–2009 (1891)	**99.89**	0.05	99.95	100		**99.84**	99.79	99.79	99.79			3.01		**99.9**		**99.26**
Avian (159)	4.4	94.97	100	100	98.11	1.26	99.37	100	100			0.63		1.26		1.26
Swine (332)	20.78	67.47	98.49	99.4	96.39	1.51	91.57	100	99.7	0.9	0.3	**33.13**	3.31	0.6	0.6	2.41

The human seasonal H1N1 viruses were separated into seven groups according to their glycosylation site patterns. The conservation of potential glycosylation sites was shown as percentage (‘%’ had been omitted) and the glycosylation site alteration was highlighted in bold. The numbers of corresponding strains used for the analysis were given in the brackets.

### Alteration of glycosylation site numbers on HA and NA in the evolution of human seasonal influenza viruses

To trace the history of the acquisition of new glycosylation sites on both HA and NA of the human seasonal H1N1 viruses, we systematically investigated the glycosylation site patterns in the HA and NA of human strains over the last 92 years (from 1918 to 2009). According to the glycosylation site patterns that would result from the acquisition, loss or positional conversion of potential glycosylation sites, the evolution of both HA and NA glycosylation for human seasonal H1N1 virus can be separated into several phases (Table S1, Table S2). Note that a new set of human seasonal strains would be grouped when a new glycosylation site appeared or an existed glycosylation site disappeared (conversion of the glycosylation site <5%). And the seasonal strains isolated in 1933 (including the WSN strains) were not included in the further analysis, as they might be selected for changes following extensive in vitro and in vivo passage in the laboratory for mouse neurovirulence [35], [36].

For HA (Table 1), five potential glycosylation sites at positions 27, 28, 40, 304 and 498 on the stalk were strictly conserved in all human strains. The potential glycosylation site 557 was also highly conserved, but it might not be glycosylated as this site was located at the intracellular region of HA. Pandemic 1918 A/H1N1 viruses had only one potential oligosaccharide at location 104 of the globular head of HA. However, the 1934–1936 isolates (represented by A/Phila/1935) lost glycosylation site 104 and acquired two new glycosylation sites at locations 286 and, occasionally, 144. The 1940–1949 isolates (represented by A/AA/Huston/1945) reacquired glycosylation site 104 and acquired an additional glycosylation site at location 179. From 1950 to 1957, the isolates (represented by A/Albany/1618/1951) lost glycosylation site 179 and gained three more glycosylation sites at locations 90, 172 and 177. When seasonal H1N1 viruses reappeared in 1977, the isolates lost glycosylation site 90. In 1986, glycosylation site 142 replaced glycosylation site 144 and a new glycosylation site at location 71 appeared. Then, glycosylation site 172 was lost in 1988, and glycosylation site 286 disappeared in 1998. Therefore, the seasonal A/H1N1 strains recently circulating in humans have two more glycosylation sites (sites 142 and 177) on the top of the HA head and one more (site 71) on the side of the HA head than the pandemic 1918 strains.

For NA (Table 2), there were seven potential glycosylation sites (four on the stalk and three on the head) on the NA of the pandemic 1918 isolates. The 1934 isolates, just like the 1933 strains (Table S2), were a little abnormal which may resulted from their propagation in eggs prior to sequencing [35], [36]. The 1935 isolates added one glycosylation site at location 44 and the 1936–1947 isolates added one glycosylation site at location 365. In 1948, the viruses lost glycosylation site 50 and gained a new glycosylation site at location 455. Glycosylation site 68 was replaced by glycosylation site 70 in 1980. An addition glycosylation site at residue 434 appeared in 1986 and glycosylation site 365 was lost in 1988. From then on, the human seasonal A/H1N1 strains appeared two new glycosylation sites on the head of the NA compared with the pandemic 1918 strains, besides one new glycosylation site at residue 386, which appeared in more than half of the strains isolated in 2000 (Table S2).

Based on the analysis above, the overall trends of glycosylation site alteration in the evolution of human seasonal A/H1N1 viruses were that the glycosylation site numbers increased continuously before 1950 for both HA and NA, then the numbers almost stayed constant, but positional conversion became the dominating mode.

### Alteration of glycosylation site locations on HA and NA in the evolution of human influenza viruses

**Figure 1:**
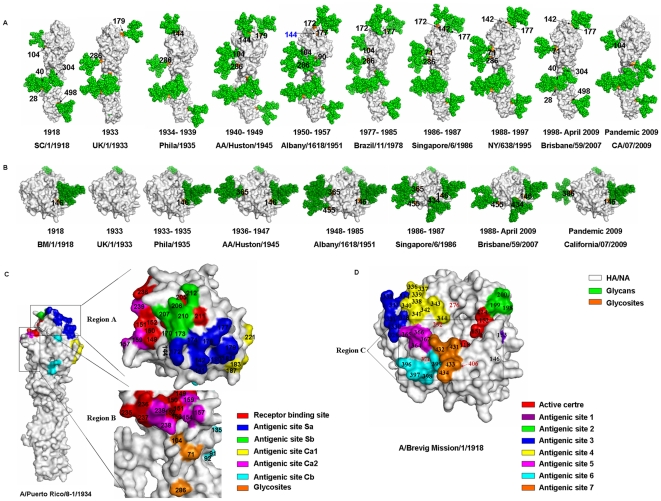
Structural overviews of the HA and NA monomers for human influenza H1N1 viruses. Each represented a unique glycosylation site pattern on HA or NA. (A) The monomers of HA with glycans attached at the potential glycosylation sites for representative human influenza H1N1 viruses from 1918 to 2010. (B) The monomers of NA with glycans attached at the potential glycosylation sites for representative human influenza H1N1 viruses from 1918 to 2010. (C) A monomer of HA with the location of the receptor-binding site (RBS) and the five antigenic sites [48], [49]. Regions A and B are important regions with frequently altered glycosylation sites. (D) A monomer of NA with the location of the enzyme active site and the seven antigenic sites surrounding the enzyme active [40], [50]. Region C is an important glycosylation region surrounding the enzyme active site. The amino acid locations are numbered according to the HA of SC 1918 and NA of BM 1918 numbering, respectively.

**Figure 2:**
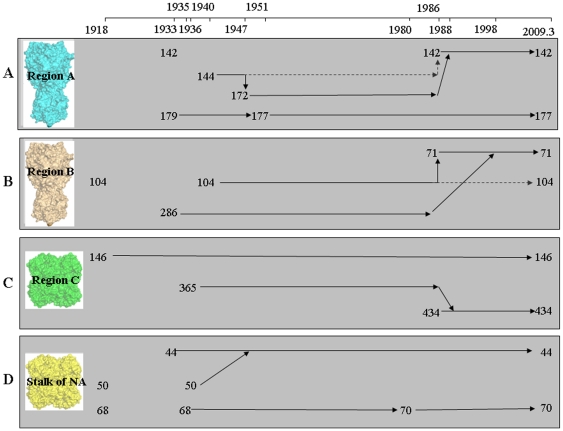
The alteration processes of glycosylation sites in some important regions of HA and NA. (A) The alteration process of glycosylation sites in region A (located at the receptor binding domain) of HA. (B) The alteration process of glycosylation sites in region B (located at the vestigial esterase domain) of HA. (C) The alteration process of glycosylation sites in region C (located around the enzymatic active site) of NA. (D) The alteration process of glycosylation sites on the stalk of NA. The dotted lines represented the superficial alterations based on genome-based analysis, while the corresponding full lines illustrated the possibly alteration processes after further analysis by homology modeling and *in silico* protein glycosylation.

To further analyze the positional conversion of glycosylation sites on HA and NA in human H1N1 viruses, we modeled the HA and NA proteins from some representative human strains using known structures of homologous proteins as a model and then added complex glycans to all accessible glycosylation sites on the modeled proteins. The results showed that although most of the potential glycosylation sites on HA (Figure 1a) and NA (Figure 1b) were occupied by glycans, the glycosylation of potential glycosylation sites 27, 144 and 104 on HA was obstructed by the steric hindrance of surrounding amino acid residues and the glycans attached at glycosylation sites 28, 172 (represented by A/Albany/1618/1951) and 71 (represented by A/NY/638/1995), respectively. In addition, the fact that potential glycosylation site 27 on HA couldn't be glycosylated had been confirmed in H5N1 influenza virus by mass spectrometric analysis previously [7]. The 3D structures of glycosylated HA and NA revealed that the glycosylation site alteration, especially positional conversion, mainly located at two important regions of the HA head, termed region A and region B (Figure 1c), and one region of the NA head, termed region C (Figure 1d). The possibly positional conversion of glycosylation sites in regions A and B of HA as well as region C and stalk region of NA was shown in Figure 2.

Region A locates at the top of the HA head, including glycosylation sites 142, 144, 172, 177 and 179 (Figure 2a). It has been confirmed that the glycans at glycosylation sites 142 and 177 could effectively block an immune reaction from antibodies directed against both pandemic 1918 and 2009 strains [26], we speculated that the glycans at glycosylation sites 144, 172 and 179 might have the same function. The glycans at glycosylation sites 142 and 177 may shield the antigenic site Sa more effectively because they are located at the center Sa, while glycosylation sites 172 and 179 are at the edge of the antigenic site and glycosylation site 144 is just adjacent to Sa (Figure 1c).

Region B, which belongs to the vestigial esterase domain and may have played a role as a fusion protein that inserted the virus into an ancestral membrane before giving rise to the modern version of HA [37], includes glycosylation sites 71, 104 and 286 (Figure 2b). One of the functions of the glycan at glycosylation site 104 is likely to shield the antigenic site Ca2, but the glycosylation of this site might be obstructed by the steric hindrance of surrounding amino acid residues. This obstruction could be weakened greatly when glycosylation occurred at glycosylation site 71(Figure 1c). In addition, the glycan at glycosylation site 71 could also shield Ca2 as well as glycosylation sites 104 and 286. Thus, it is not surprising that glycosylation site 71 replaced the glycosylation sites 104 and 286 eventually.

There are three glycosylation sites (glycosylation sites 146, 365 and 434) around the enzymatic active site of each NA monomer (Region C in Figure 1d and Figure 2c). Glycosylation at glycosylation site 146 of NA might be necessary for the function of NA and even for the survival of the virus because glycosylation site 146 was highly conserved in almost all H1N1 strains regardless of the host (Table 2). Glycosylation sites 365 and 434 belonged to antigenic sites 5 and 7, respectively (Figure 1d). Therefore, the glycans attached to both sites could increase the resistance of the virus to host immunity and/or regulate the activity of NA. Besides, glycosylation site 434, like glycosylation site 146, is at the subunit interface. So the glycan at glycosylation site 434 may also have a function of stabilizing the NA tetramer, which should be one of the important reasons that glycosylation site 434 replaced glycosylation site 365 entirely in 1988.

Positional conversions of glycosylation sites also occurred in the NA stalk region of the human seasonal influenza viruses (Figure 2d). The stalk region of NA is probably the most exposed and vulnerable region to protease attacks, as NA is frequently released from viral particles through proteolytic cleavage of this region [22], [38], [39], [40]. The glycans at glycosylation sites 44 and 70 may be more effectively than at glycosylation sites 50 and 68 in protecting the NA stalk avoiding the effects from human proteases.

It can be concluded from the analysis above that glycosylation site alteration occurred more frequently on HA than on NA, and more frequently on the top of the HA head than on the side of the HA head. Positional conversion of glycosylation sites, especially the positional conversion occurred at many new glycosylation sites acquired in the early stages of the evolution of influenza virus (such as glycosylation site 365 on NA convert to site 434, and glycosylation sites 144 and 179 on HA convert to sites 142 and 177, respectively), implied the significance of location of glycans on both HA and NA for maximization of their biological functions in the evolution of human influenza A/H1N1 viruses.

### Conservation of glycosylation sites on HA and NA of human seasonal influenza viruses

Generally, the speed with which a new mutant strain overtakes the original strain indicates the new strain's superiority. For the glycosylation site alteration on HA and NA of influenza A/H1N1 virus, though this analysis is hindered by the limited number of sequences available until 1995, the overall tendency was that a new mutant strain with positional conversion of glycosylation sites on HA and NA could more rapidly overtakes the original strain than a new mutant strain with simple acquisition of new glycosylation sites on HA and HA (Table 1 and 2). For example, the conservation levels of glycosylation sites 144 and 172 on HA and glycosylation site 365 on NA were low when they first appeared, and then it gradually increased. While glycosylation sites 142 and 71 on HA and glycosylation sites 434 on NA were highly conserved since they first appeared, This implied that positional conversion of glycosylation sites might be a more effective mode of glycosylation site alteration for the evolution of influenza A/H1N1 viruses. The relatively low frequency of positional conversion of glycosylation sites in the later stages of evolution also supported this conclusion.

### Glycosylation site alteration on HA and NA of influenza viruses from other hosts

In recent decades, both classical swine influenza and triple-reassortant swine influenza viruses have occasionally been isolated from humans [41], [42], such as during the 1976 outbreak in Fort Dix, New Jersey [41], [43]. Our analysis showed that these swine influenza strains isolated from humans had identical patterns for potential glycosylation sites on HA and NA as the pandemic 1918 and 2009 strains, but completely different patterns from the human seasonal strains circulating during the same periods (Figure S1 and S2, Tables S1 and S2).

For further analysis of the glycosylation site alteration in influenza A/H1N1 viruses from other hosts, we further analyzed the patterns of the potential glycosylation sites on HA and NA of influenza A/H1N1 viruses isolated from avian and swine (Table 1, Table 2). The different glycosylation site patterns on HA and NA among human, avian and swine influenza A/H1N1 viruses implied the different evolutionary processes of influenza H1N1 viruses in different hosts, and the different evolutionary paths of H1N1 viruses implied different immune systems and selective pressures against influenza viruses in different hosts. Based on the analysis above, it could be concluded that avian possessed the lowest selective pressure, while human possessed the highest selective pressure against influenza A/H1N1 virus. All influenza A/H1N1 viruses isolated from other mammals (including cat, ferret and giant anteater) possessed the same glycosylation site patterns as the human seasonal and pandemic influenza viruses and depended on the predominant strains circulating in humans at the time. But these data were not included in the study on account of insufficiency of HA and NA sequences available for statistical analysis (Table S1 and S2).

## Discussion

Genome-based approaches were typically used when investigating changes in glycosylation, as well as when predicting function, due to the great simplicity of these measurements [6], [25], [27], [44]. Such studies were based upon locating the sequon in the amino acid sequence predicted by the viral RNA. This method was based on the assumption that all potential *N-*glycosylation sites were occupied. Unfortunately, the amino acid sequence is only one determinant of glycosylation because the location of glycosylation sites and the host environment also have a strong effect on glycosylation [8], [29], [45], [46]. Moreover, the amino acid sequence of glycoproteins alone is not sufficient to obtain the location information of glycosylation sites in the 3D structures of glycoproteins. In this study, a series of bioinformatics tools were used to maximize the reliability of this strategy. First, all of the sequons were found from 2,773 full-length amino acid sequences of HA and 3,249 full-length amino acid sequences of NA from influenza H1N1 viruses over a 93-year period and were assumed to be potential glycosylation sites. Then, the structures of representative HA and NA proteins from human influenza A (H1N1) viruses were modeled, including their different patterns of potential *N-*glycosylation sites. Finally, glycans were added *in silico* onto each glycosylation site using the Glyprot server to confirm that these potential glycosylation sites could be glycosylated, and the structures of the obtained glycoproteins were further used to determine the location of glycosylation sites.

Our results showed that two modes of protein glycosylation site alteration were involved in the evolution of human seasonal influenza viruses. The first mode was to increase the number of glycosylation sites. This mode was obvious and have been reported many times before in both human H1N1 and H3N2 viruses [6], [17], [25], [26], [27], [44]. The other mode was the positional conversion of glycosylation sites (also called glycosylation site substitution), which occurs when the acquisition of a new glycosylation site in the new strain is accompanied by the loss of an existing glycosylation site. The acquisition and loss of glycosylation sites may not occur simultaneously and the acquired glycosylation sites may also not adjacent to the lost glycosylation sites in the primary structures of the proteins, as some of the exchanges occurred over an intermediate time and/or distance frame (for instance, the conversion of glycosylation site 286 on HA to glycosylation site 71). This hides the second mode and makes it more likely to be neglected in the evolutionary analysis of influenza viral protein glycosylation [25]. Our results showed that the alteration of glycosylation sites by the first mode mainly occurred with high frequency in the early stages of viral evolution (1918–1949 for both HA and NA of human influenza H1N1 viruses), while the second mode mainly occurred with relatively low frequency in the later stages (1950–2009 for both HA and NA of human influenza H1N1 viruses).

Our results also indicated that positional conversion of glycosylation sites might be a more effectively alteration mode. The presence of glycans on the head of HA and NA can have either positive or detrimental effects on the virus [8], [14]. While it shields antigenic sites from immune recognition, it reduces receptor affinity of HA and enzymatic activity of NA [8], [14]. So the possible process and mechanism of glycosylation site alteration in human seasonal H1N1 virus are described below. Since only few glycans attached at the antigenic sites and surrounding regions of HA and NA in pandemic H1N1 viruses, the acquisition of new glycosylation sites (increase in glycosylation site numbers) in these regions mainly play its positive role in escaping antibody recognition from hosts in the early evolution stages of seasonal strains, and thus it is necessary for seasonal virus to continue to prevail among humans. Then new immune antibodies to these seasonal strains are gradually induced in the host, decreasing the transmissibility of the virus. But if continuously added new glycans onto the antigenic sites, it would greatly reduces receptor affinity of HA and enzymatic activity of NA. Therefore, alteration of glycan location (positional conversion of glycosylation sites) became a more suitable way for evolution of H1H1 viruses. By simply changing the location of the glycans, but no new glycans added, the viruses obtained the ability of escaping immune recognition from host antibodies again. So the positional conversion of glycosylation sites may be more artful than the increase of glycosylation site numbers for the host adaptation of influenza virus.

In fact, we speculated that there might be a third alteration mode of protein glycosylation that involved in the evolution of influenza viruses: the alteration of glycan structures. This alteration could be achieved either by direct changes to the monosaccharide components and bonds of the glycans at specific glycosylation sites, or it could occur along with the positional conversion of the glycosylation sites. When a glycan transferred form one place to another, the glycan might also change to a new structure. The new structure of the glycan rather than the positional conservation of the glycosylation site might be the actual factor that made the influenza virus obtained the ability to continue to prevail among humans. However, it is still impossible to predict the structure of all of the glycans on the different glycosylation sites nowadays due to the complexity of glycans and the lack of correspondingly competent analytical techniques and tools. Therefore, this potential alteration mode was not included in this study.

Among the methods used to control the rapid spread of influenza viruses, vaccination remains the most effective [47]. A good vaccine should induce immune responses that cross-neutralize either all viruses in a subtype or, ideally, all influenza viruses. However, altered protein glycosylation, just like varied amino acid sequences, could affect the ability of antibodies to neutralize influenza viruses and thus affect the effectiveness of the vaccines [26], [29]. Therefore, the glycosylation alteration of HA and NA may need to be taken into consideration in global surveillance, vaccine production and drug design for influenza viruses.

## Supporting Information

Figure S1 **Phylogenetic tree for HA amino acid sequences of selected influenza A/H1N1 viruses.** The selected viruses include swine strains isolated from humans, representative strains isolated from swine as well as representative strains of human seasonal viruses since 1976 and the pandemic 2009 strain.
(TIF)

Figure S2 **Phylogenetic tree for NA amino acid sequences of selected influenza A/H1N1 viruses.** The selected viruses include swine strains isolated from humans, representative strains isolated from swine as well as representative strains of human seasonal viruses since 1976 and the pandemic 2009 strain.
(TIF)

Table S1 **The potential glycosylation sites of HA in H1N1 influenza viruses from different hosts.** The sequence in each unit of table represents the corresponding sequon of each site. The potential glycosylation sites were highlighted by filling the units in yellow.
(XLS)

Table S2 **The potential glycosylation sites of NA in H1N1 influenza viruses from different hosts.** The sequence in each unit of table represents the corresponding sequon of each site. The potential glycosylation sites were highlighted by filling the units in yellow.
(XLS)
