# Supplementary figures and images for: Glycosylation Site Alteration in the Evolution of Influenza A (H1N1) Viruses
Source: PLoS One. 2011 Jul 28;6(7):e22844. doi: 10.1371/journal.pone.0022844 (PMC3145772; doi:10.1371/journal.pone.0022844)

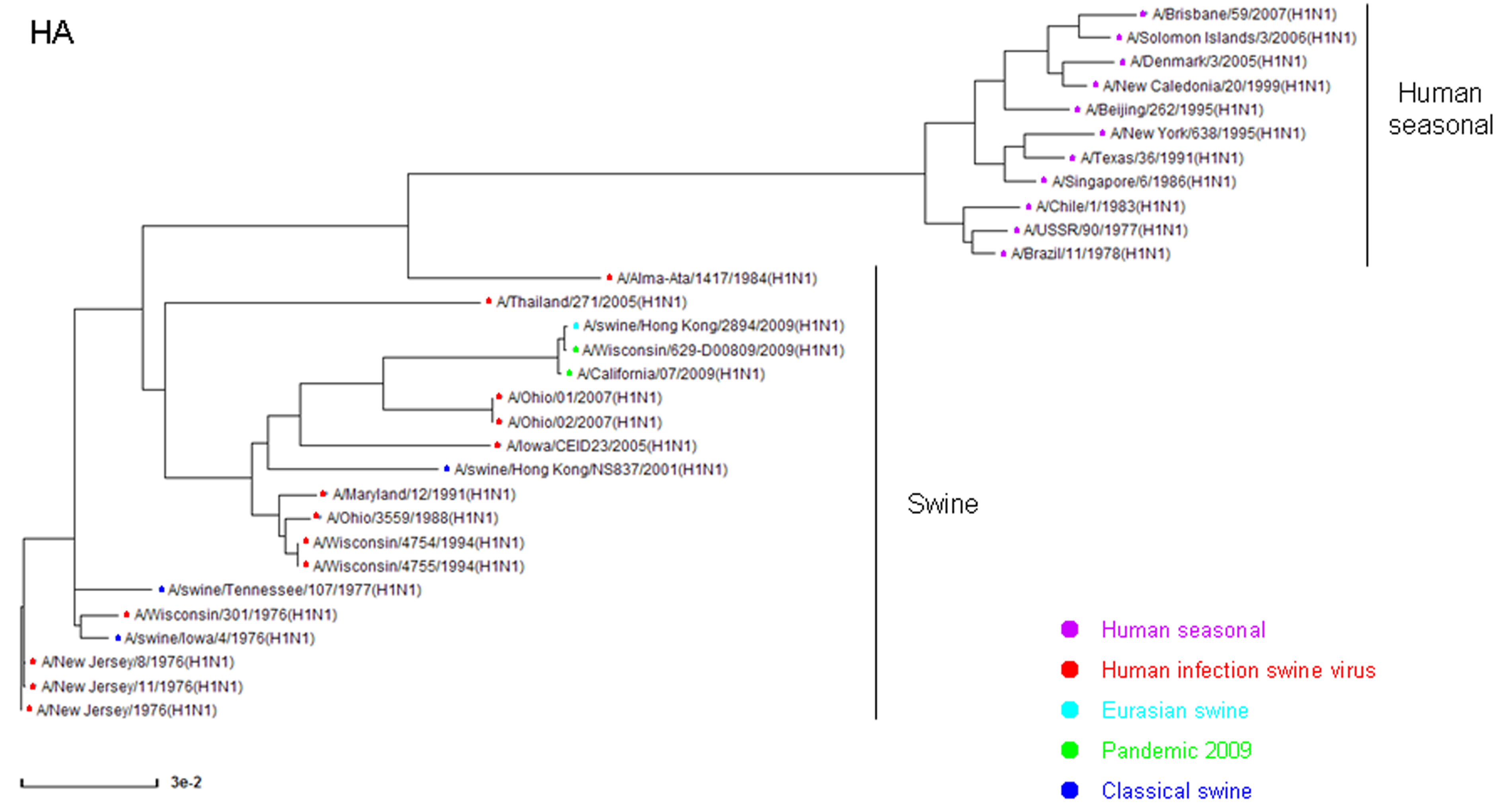

Supplement: Figure S1 — Phylogenetic tree for HA amino acid sequences of selected influenza A/H1N1 viruses. The selected viruses include swine strains isolated from humans, representative strains isolated from swine as well as representative strains of human seasonal viruses since 1976 and the pandemic 2009 strain. (TIF) [file pone.0022844.s001.tif]

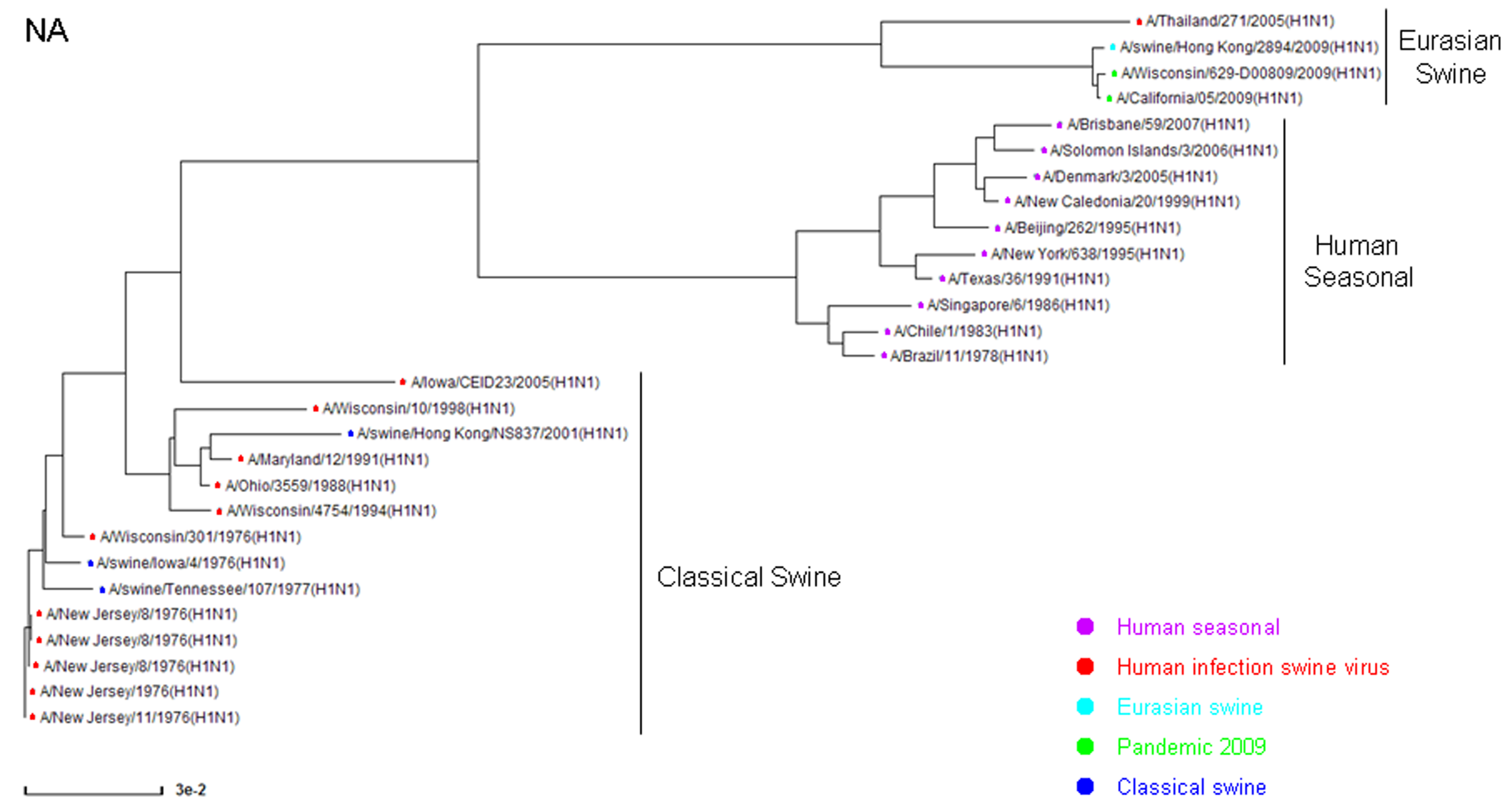

Supplement: Figure S2 — Phylogenetic tree for NA amino acid sequences of selected influenza A/H1N1 viruses. The selected viruses include swine strains isolated from humans, representative strains isolated from swine as well as representative strains of human seasonal viruses since 1976 and the pandemic 2009 strain. (TIF) [file pone.0022844.s002.tif]
